# Supplementary figures and images for: Immune Response after mRNA COVID-19 Vaccination in Lung Transplant Recipients: A 6-Month Follow-Up
Source: Vaccines (Basel). 2022 Jul 15;10(7):1130. doi: 10.3390/vaccines10071130 (PMC9318026; doi:10.3390/vaccines10071130)

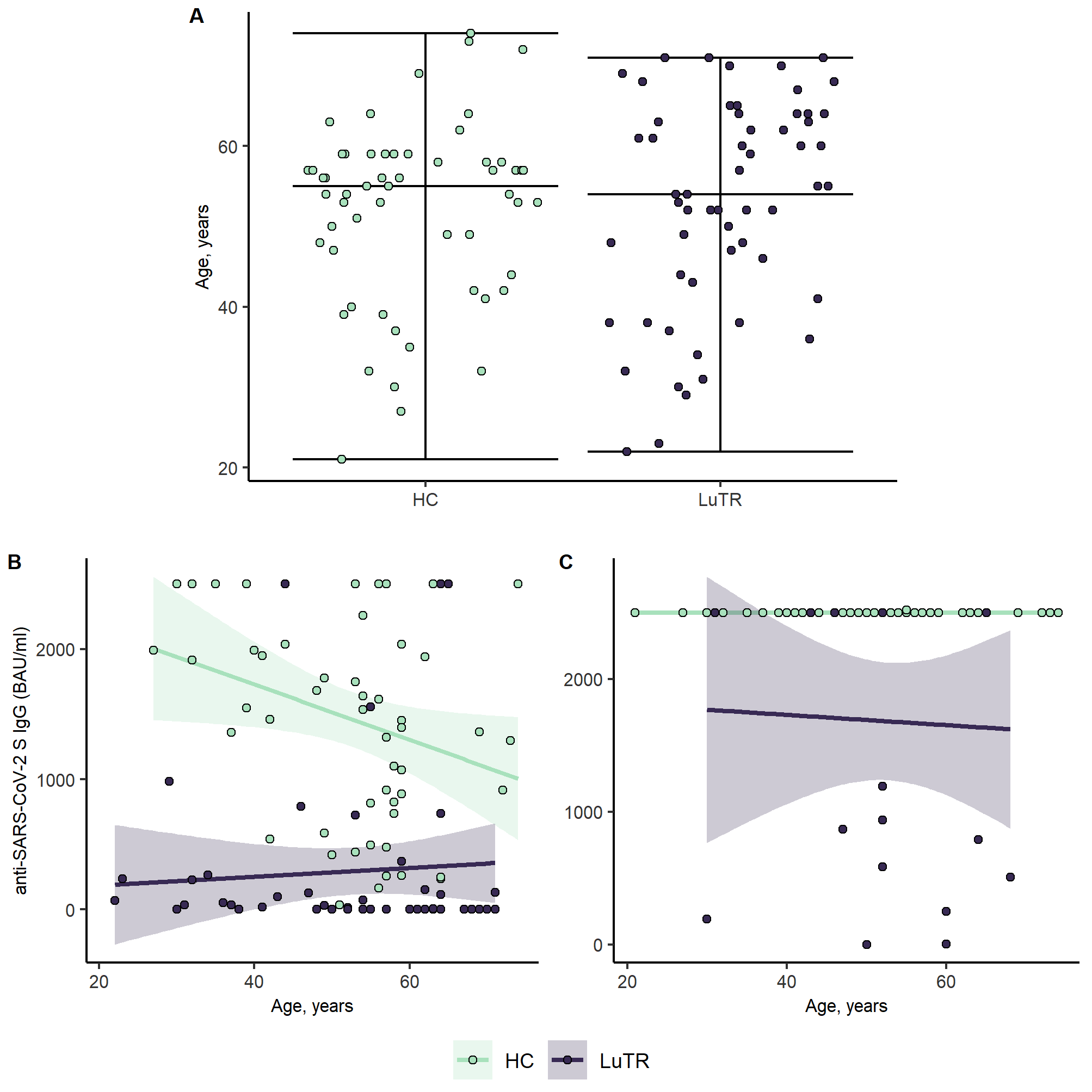

Supplement: Supplementary file 1 [file vaccines-10-01130-s001.zip › vaccines-1786372-Figure S1.tiff]
